# Supplementary material for: Subcellular and supracellular mechanical stress prescribes cytoskeleton behavior in Arabidopsis cotyledon pavement cells
Source: eLife. 2014 Apr 16;3:e01967. doi: 10.7554/eLife.01967 (PMC3985187; doi:10.7554/eLife.01967)
Supplement: Supplementary file 1. — Additional information about computational models. DOI: http://dx.doi.org/10.7554/eLife.01967.026 [file elife01967s001.pdf]

1 Supplemental information

1.1 Nonlinear Finite Element Method implementation

The implementation of the finite element method used in the simulations of cellular geometries in this paper is based on the minimization of the stain energy functional. For this purpose we write virtual work equation describing the variation of the work due to the virtual deformation as

$$\delta W = \int_\omega \sigma : \delta \mathbf{d} \, dv - \int_\omega \mathbf{f} \cdot \delta \mathbf{v} \, dv - \int_{\partial\omega} \mathbf{t} \cdot \delta \mathbf{v} \, da = 0, \tag{1}$$

where σ is Cauchy's stress tensor, $\mathbf{f}$ and $\mathbf{t}$ are body forces and tractions respectively, $\delta\mathbf{d}$ is the virtual rate of deformation tensor and $\delta\mathbf{v}$ is virtual velocity. We obtain the solution of this problem by linearization and iterative steps with respect to trial deformation solution ϕ_k by use of the Newton-Raphson procedure. The equilibrium equations linearized in the direction of increment $\mathbf{u}$ in ϕ_k can be then written as:

$$D\delta W(\phi, \delta v)[\mathbf{u}] = \int_\omega \delta\mathbf{d} : c : \epsilon \, dv + \int_\omega \sigma : [(\nabla\mathbf{u})^T \nabla \delta\mathbf{v}] \, dv + \int_\omega \mathbf{f} \cdot \delta\mathbf{v} \, dv + \int_{\partial\omega} \mathbf{t} \cdot \delta\mathbf{v} \, da, \tag{2}$$

where c is a tangent modulus of elasticity contributing to constitutive component of stress, ϵ is a strain tensor In finite element method this equation is solved by discretization of $\mathbf{u}$ and $\delta\mathbf{v}$d with respect to a local support basis for the problem (so called shape functions) which specific form depends on the choice of the finite element discretization. We have used quadrilateral shell elements with extensible director formulation which geometry can be described by the following relations:

$$\mathbf{X}(\xi_i) = \bar{\mathbf{X}}(\xi_\alpha) + \mathbf{D}(\xi_i) \tag{3}$$

$$\bar{\mathbf{X}}(\xi_\alpha) = \sum_{a=1}^{n} N_a(\xi_\alpha)\bar{\mathbf{X}}_a \tag{4}$$

$$\mathbf{D}(\xi_i) = \sum_{a=1}^{n} N_a(\xi_\alpha) z_a(\xi_3)\bar{\mathbf{D}}_a \tag{5}$$

$$z_a(\xi_3) = N_+(\xi_3) z_a^+ + N_-(\xi_3) z_a^- \tag{6}$$

$$N_+(\xi_3) = \frac{1}{2}(1+\xi_3), \quad N_-(\xi_3) = \frac{1}{2}(1-\xi_3), \tag{7}$$

where Latin and Greek indices are assumed to span from 1 to 3 and from 1 to 2 respectively. The 3D position of the body particle $\mathbf{X}$ is described by the point on the reference surface $\bar{\mathbf{X}}$ and the vector $\mathbf{D}$ called the director. The reference surface and the director are described by the two dimensional shape functions N_a and nodal values $\bar{\mathbf{X}}_a$ and $\bar{\mathbf{D}}_a$. The function z_a describes the thickness of the element in terms of the distance from the reference surface to the bottom and top surfaces. The director vector in deformed configuration is not required to be of unit length and takes into account the changes in the thickness.

1.2 Material description

To represent a pavement cell wall material we use hyperelastic transversely isotropic model. Hyperelasticity means that the stress arises solely from the deformation (and not how this deformation arises) and that there exist a scalar strain energy function W from which the stress can be derived at any point. The second Piola-Kirchhoff stress $\mathbf{S}$ and material elasticity $\mathcal{C}$ tensors can be then obtained as derivatives of the strain energy with respect to strain tensor $\mathbf{E}$

$$\mathbf{S} = \frac{\partial W}{\partial \mathbf{E}}, \tag{8}$$

$$\mathcal{C} = \frac{\partial \mathbf{S}}{\partial \mathbf{E}}. \tag{9}$$

The transverse isotropy means that there is a single direction in which the properties of the material are different. This direction in the model is represented by the vector $\mathbf{a}$ which introduces the dependence of the stain energy on the preferred direction. In our simulations we have assumed that this direction is dynamically controlled by the cell and set it at each step of the simulation to the direction of the maximal principal stress in each element. With the assumptions of transversely isotropic elasticity the strain energy can be expresses as a function of the set of invariants of the right Cauchy-Green deformation tensor $\mathbf{C}$

$$W = F_1(I_1, I_2, I_3) + F_2(I_4, I_5) + F_3(I_1, I_2, I_3, I_4, I_5), \tag{10}$$

where $I_1 = tr\mathbf{C}$, $I_2 = \frac{1}{2}[(tr\mathbf{C})^2 - tr\mathbf{C}^2]$, $I_3 = det\mathbf{C} = J^2$, $I_4 = \mathbf{a}\cdot\mathbf{C}\cdot\mathbf{a} = \lambda^2$, $I_5 = \mathbf{a}\cdot\mathbf{C}^2\cdot\mathbf{a}$. The function F_1 describes the behavior of the isotropic matrix part of the material, F_2 corresponds to the response of the fibers and F_3 represents the interaction between fibers and and matrix. In our case the fibers represented not only cellulose microfibrils, but also their tethering by xyloglucans and other components of the wall and were modeled in average sense by the function F_2 satisfying relations for $\lambda^2 = I_4$ being the fiber stretch

$$\begin{aligned}
\lambda\frac{\partial F_2}{\partial \lambda} &= 0, \ \ \lambda < 0, \\
\lambda\frac{\partial F_2}{\partial \lambda} &= C_3\left(exp(C_4(\lambda - 1)) - 1\right), \ \ \lambda < \lambda^*, \\
\lambda\frac{\partial F_2}{\partial \lambda} &= C_5\lambda + C_6, \ \ \lambda \geq \lambda^*.
\end{aligned}$$

The λ^* corresponds to the the stretch at which the initial exponential response becomes linear and in the simulations it was assumed $\lambda^* = 1.02$. The C_3 is a coefficient of exponential stress, C_4 gives the rate of attaining linear response, C_5 is a modulus of fibers and C_6 is calculated by the constrain of the continuity of a fiber stress at λ^*. As a material of the matrix we have tested Saint Venant–Kirchhoff model with strain energy function given with respect to deviatoric invariants $\tilde{I}_1$, $\tilde{I}_2$ by

$$F_1(I_1, I_2, I_3) = 0.5(\lambda + 2\mu)\left(0.5(\tilde{I}_1 - 3)\right)^2 - 0.5\mu(\tilde{I}_2 - 2\tilde{I}_1 + 3) + 0.5\kappa(J-1)^2, \qquad (11)$$

where the initial bulk modulus can be calculated from Lame parameters $\kappa = \lambda + 2\mu/3$, and the Mooney-Rivilin model with analogical function in the form

$$F_1(I_1, I_2, I_3) = C_1(\tilde{I}_1 - 3) + C_2(\tilde{I}_2 - 3) + 0.5\kappa(J-1)^2. \qquad (12)$$

Both of the matrix models gave qualitatively equivalent results thus we presented the results with easier to interpret from material coefficients point of view Saint Venant–Kirchhoff model.
